# Amisulpride withdrawal dyskinesia: a case report

**DOI:** 10.1186/s12991-017-0148-0

**Published:** 2017-06-14

**Authors:** Yu-Chi Lo, Ying-Chieh Peng

**Affiliations:** 1 Ministry of Health and Welfare Kinmen Hospital, No. 2, Fuxing Rd., Jinhu Township, Kinmen County, 89142 Taiwan; 2 Bali Psychiatric Center, No. 33, Huafushan, Bali Dist., New Taipei City, 24936 Taiwan

**Keywords:** Amisulpride, Antipsychotics, Withdrawal, Dyskinesia, Side effects, Schizophrenia, Case report, Abnormal involuntary movement

## Abstract

**Background:**

The effects of antipsychotic drug withdrawal have been inadequately studied. Case reports have described dyskinesia occurring in patients with several antipsychotics withdrawn, but studies on amisulpride withdrawal dyskinesia are lacking.

**Case presentation:**

A 63-year-old man, who was diagnosed with schizophrenia at age 49, received amisulpride treatment since age 62. The dosage of amisulpride was reduced from 200 to 50 mg/day because of occurrence of akathisia during one admission. Severe withdrawal dyskinesia, mixed with dystonia and akathisia, was noted immediately after the dosage reduction. The abnormal involuntary movement showed improvement 2 weeks later when the dosage was increased to 100 mg/day.

**Conclusions:**

Withdrawal dyskinesia and other abnormal involuntary movements could be noted in a patient with reduction of amisulpride dosage or sudden termination. Withdrawal dyskinesia may subsequently lead to persistent tardive dyskinesia. Therefore, withdrawal-emergent dyskinesia should be promptly identified, and appropriate medical interventions should be given early.

**Electronic supplementary material:**

The online version of this article (doi:10.1186/s12991-017-0148-0) contains supplementary material, which is available to authorized users.

## Background

Several chronic schizophrenic patients require antipsychotic maintenance treatment. Discontinuation of antipsychotics in patients with schizophrenia often leads to increased risk of psychotic relapse. However, the effects of antipsychotic drug withdrawal have not been adequately studied. A recent investigation indicated that patients experienced a range of discontinuation symptoms, including physical, cognitive, emotional, psychotic or sleep-related disturbances [1]. Therefore, more attention should be paid to discontinuation symptoms to identify the discomfort suffered by patients withdrawn from antipsychotics. Case reports have described dyskinesia occurring in patients with several antipsychotics withdrawn, but studies on amisulpride withdrawal dyskinesia are lacking.

## Case presentation

A 63-year-old man was diagnosed with schizophrenia at age 49 and had been treated at the outpatient department of Songde Branch of Taipei City Hospital since the diagnosis. His symptoms included delusional jealousy, persecutory delusion, irritability, self-destructive behaviour and self-talk. Sulpiride 600 mg/day was prescribed but he quit the outpatient follow-up 2 months later. From age 50, he had an average daily consumption of four standard drinks of alcohol. Drinking worsened his impulse control and caused him to get into conflicts with his neighbours. He had been admitted to the acute psychiatric ward of Songde Branch of Taipei City Hospital three times since age 55. Brain computed tomography was performed that showed mild bilateral frontal lobe atrophy, but no other significant abnormal findings were identified. Benzodiazepine and thiamine were given whenever he was admitted for the prevention of Wernicke’s encephalopathy and alcohol withdrawal symptoms. Neither acute confusion state nor obvious amnesia was noted during admission.

Risperidone 4 mg/day was used at first during admission for about 1 month with significant improvement in his psychotic symptoms. Unfortunately, the side effect dysphasia occurred and the patient developed aspiration pneumonia. Therefore, risperidone treatment was ceased and quetiapine was prescribed with gradual increase in dosage to about 550 mg/day. However, the treatment efficacy was not satisfactory in this patient.

At age 62, amisulpride at a dosage of 400 mg/day was prescribed instead in the outpatient department. After 6 months, obvious akathisia was noted, and he was transferred to Bali psychiatric centre for further treatment. The amisulpride dosage was tapered to 200 mg/day. His psychotic symptoms remained stable, but the side effects persisted, and amisulpride was further reduced to 50 mg/day gradually. His akathisia showed improvement, but he began to suffer from other forms of abnormal involuntary movements comprising dyskinesia mixed with dystonia and akathisia. The involuntary movements involved his trunk, head and neck, and four limbs. He kept twisting his body and head around or back and forth (Additional file 1: Video 1), which may be the dyskinesia symptoms mixed with the cervical dystonia. Further, the feeling of inner restlessness and a compelling need to be in constant motion meant that akathisia played a role in his abnormal involuntary movements. Meanwhile, the stepping movement could also be observed when he was made to sit (Additional file 1: Video 1). According to the patient and his family’s report, the abnormal involuntary movements, which were subsequent to the dosage reduction of amisulpride, never occurred previously.

The symptoms persisted for 2 weeks until amisulpride was increased to 100 mg/day. He still had slightly involuntary movement of his head and body but with much declined severity (Additional file 2: Video 2). Besides the adjustment of antipsychotic dosage, we used propranolol 10 mg twice a day for treatment of his akathisia and biperiden 0.5 mg twice a day for some dystonia-like symptoms. Further, lorazepam (0.5 mg twice a day) was prescribed for supplementary treatment of akathisia and dyskinesia for about 2 weeks. After discharge, the patient got relapsed about 1 year later with poor treatment adherence. The abnormal involuntary movements were still noted, although very mild, even though he did not take any psychotropic medications for about 3 months.

## Discussion

Withdrawal-emergent syndrome was first described in 1973 by Polizos et al. [2] as choreatic movements in children after abrupt discontinuation of an antipsychotic drug after long-term use. The symptoms usually manifest during the first few days or weeks after discontinuation of dopamine receptor-blocking agents. It is believed to be caused by a temporary hyper-dopaminergic state in the basal ganglia following the discontinuation of dopamine receptor-blocking drugs [3]. Further, the most prominent theories on tardive dyskinesia pathogenesis could account for the occurrence of withdrawal dyskinesia. The theories indicate that chronic exposure to neuroleptics results in D2 receptor upregulation with postsynaptic dopamine receptor supersensitivity. D2 receptors are inhibitory receptors expressed on medium-spiny neurons that project onto the indirect pathway; hence, their hypersensitivity can result in disinhibition of the globus pallidus internus and the subthalamic nucleus, producing a variety of hyperkinetic movement disorders [4].

Case reports have described dyskinesia occurring after withdrawal of different antipsychotics, including chlorpromazine and fluphenazine [5], mesoridazine [6], haloperidol [7, 8], risperidone [9–12], aripiprazole [12–14] and clozapine [15]. However, there is no literature on amisulpride withdrawal dyskinesia. The present case illustrated that dyskinesia does occur in patients withdrawn from amisulpride.

Gardos et al. [3] classified the dyskinetic movements seen during antipsychotic treatment into the following three distinct categories: tardive dyskinesia, withdrawal dyskinesia and covert dyskinesia. Withdrawal dyskinesia, in contrast to tardive dyskinesia, is self-limited, whereas covert dyskinesia refers to a dyskinesia that develops in response to dose reduction or drug discontinuation, does not disappear spontaneously and may become permanent. It has been suggested that if withdrawal dyskinesia does not subside within 12 weeks, it is likely to be a covert dyskinesia [3].

Some studies have indicated that withdrawal-emergent movements in subjects who are not yet diagnosed with tardive dyskinesia (TD) may represent an early phase of neurological dysfunction on a continuum ultimately leading to persistent TD [16–18]. Therefore, antipsychotic withdrawal-emergent dyskinesia should be promptly identified and appropriate medical interventions should be taken as early as possible. These interventions may include minimising the dosage of medication, switching from a conventional to an atypical antipsychotic and prescribing quetiapine or clozapine instead [18, 19], although clozapine-withdrawal dyskinesia has also been reported [15]. In addition, other less studied medications that may provide various degrees of symptomatic improvement in TD could be considered to alleviate withdrawal dyskinesia. These medications include amantadine that acts as a glutamate receptor-blocking agent [20] and gamma-aminobutyric acid (GABA) agonistic medications such as benzodiazepines [21], lithium [22], vitamin E [23], melatonin [24] and zolpidem [25]. Nevertheless, all these observations are from small open-label studies and case reports.

As for the present case described here, he got relapsed and was admitted to our acute psychiatric ward 1 year after last discharge. He did not take any psychotropic medications for about 3 months; however, a mild form of truncal and neck dyskinesia could still be observed. This was probably consistent with the ‘covert dyskinesia’ mentioned by Gardos et al. [3].

There is a limitation of the case report. Neurodegenerative process at the age of 63 would represent a potential cause regarding the development of dyskinesia; however, we did not arrange dopamine imaging for him. The reason was that the neurological examinations at admission did not show focal neurological signs or obvious abnormal involuntary movements except akathisia. The abnormal involuntary movements were noted immediately after the dosage reduction, and it never occurred previously. Therefore, other neurodegenerative diseases were not favoured, and the dopamine imaging was not performed. Thus, neurodegenerative disease induced dyskinesia could not be ruled out completely.

## Conclusion

Withdrawal dyskinesia could be noted in patients with reduction in amisulpride dosage or sudden termination. Previous case reports included withdrawal of both typical and atypical antipsychotics. Some studies indicated that antipsychotic withdrawal-emergent movements in patients may subsequently lead to persistent TD. Therefore, withdrawal-emergent dyskinesia should be promptly identified, and appropriate medical interventions should be given as early as possible.

## Additional files

**Additional file 1: Video 1.** The abnormal involuntary movements after withdrawal of amisulpride.

**Additional file 2: Video 2.** The abnormal involuntary movements showed improvement when the dosage of amisulpride was increased.

## Abbreviation

TD: tardive dyskinesia
